# Another milestone for the Annals of Indian Academy of Neurology

**DOI:** 10.4103/0972-2327.58270

**Published:** 2009

**Authors:** Sanjeev V. Thomas

**Affiliations:** Editor, Annals of Indian Academy of Neurology, Departments of Neurology, Sree Chitra Tirunal Institute for Medical Sciences and Technology, Trivandrum, India. E-mail: editor@annalsofian.org

This is a landmark issue in the history of the Annals of Indian Academy of Neurology. I am delighted to announce that AIAN is now indexed in the PubMed. You can view citations from this journal like any other indexed journal at the PubMed search page. Please note that it may take a few weeks, before all the articles from recent issues of the AIAN are uploaded on the domain. We do appreciate the support and encouragement that we received in this endeavor from the Academy, readers, contributors, reviewers, and the publishers in this regard. We do hope that indexing with the PubMed, Science Citation Index, and other indexing databases would increase the visibility of articles published in this journal and encourage more authors to contribute their manuscripts to us.

This issue of the journal is devoted to multiple sclerosis. Professor Rammohan Kottil from Ohio State University, Columbus, had kindly agreed to our request to be the guest editor for this theme-based issue. He has done an excellent job in soliciting articles from scientists and clinicians with vast experience in their respective areas of interest. We are particularly thankful to Professor Kottil for his untiring efforts, despite various other commitments and temporary ill health, to see that the issue has come out very well. We hope that the readers would find these articles very helpful. With this issue onwards, we are advancing the publication time from the end of the quarter to the beginning of each quarter. This would bring each issue to the reader earlier than before and also increase the opportunity for more citations for the published articles. Let me take this opportunity to wish all the members of the Academy and the readers a Merry Christmas and Happy New Year.

